# Case Report: Atypical visual presentation caused by a large convexity meningioma—a nerve stretching and stalk indentation effect

**DOI:** 10.3389/fsurg.2024.1399163

**Published:** 2024-07-18

**Authors:** Chia-Hsun Chiu, Ko-Ting Chen

**Affiliations:** ^1^ Department of Neurological Institute, Taichung Veterans General Hospital, Taichung, Taiwan; ^2^ Department of Neurosurgery, Chang Gung Memorial Hospital at Linkou, Taoyuan, Taiwan; ^3^ School of Medicine, Chang Gung University, Taoyuan, Taiwan; ^4^ Neuroscience Research Center, Chang Gung Memorial Hospital at Linkou, Taoyuan, Taiwan

**Keywords:** meningioma, optic nerve injury, pituitary stalk, visual field defect, optic nerve swelling

## Abstract

Visual field defects are commonly present in patients with brain tumors, particularly due to direct compression on the optic apparatus. However, there are instances where brain tumors, despite not directly compressing the optic pathway, can still cause visual symptoms, albeit rarely reported but not uncommonly observed. These mechanisms are thought to be associated with increased intracranial pressure (IICP). We report a case of a 32-year-old man who presented with right blurred vision and was diagnosed with a right convexity meningioma. Upon reviewing his magnetic resonance images, we hypothesized that the indentation of the pituitary stalk on the optic chiasm and the stretching of the optic nerve, combined with a focal effect of IICP, could be responsible for his atypical visual field defect.

## Introduction

Pituitary adenoma, craniopharyngioma, posterior fossa tumor, and meningioma are among the most commonly reported intracranial tumors with visual manifestations (1). Up to 67.9% of patients with brain tumors presented with visual complaints at their initial presentation, according to one retrospective series (2). The most commonly reported ophthalmic manifestations were visual blur and visual field defects, including quadrantanopia, homonymous hemianopia, and bi-temporal hemianopia (1, 3, 4). The optic pathway originates from the retina of the eye and passes through the optic nerve. The optic nerve fibers from the nasal side then decussate to the contralateral side at the optic chiasm. Beyond the optic chiasm, the optic nerve becomes the optic tract and connects to the lateral geniculate nucleus of the thalamus. Subsequently, the optic fibers become the optic radiations and ultimately connect to the primary visual cortex in the occipital lobe (5). The ophthalmic symptoms vary depending on the location of the tumor. A pre-chiasmal tumor compressing on the ipsilateral optic nerve fibers would lead to a defect in the ipsilateral visual field. A tumor located in the sellar region of the brain with compression on the optic chiasm, particularly the bilateral nasal side optic fibers, would cause bi-temporal hemianopia, whereas a post-chiasmal tumor would cause homonymous hemianopia (6).

However, some brain tumors do not directly compress on the optic pathway yet still cause visual symptoms (7). This may be related to the increased intracranial pressure (IICP) induced by the tumor. Modern theories propose that the high intracranial pressure increases the cerebrospinal fluid (CSF) pressure surrounding the optic nerves and disrupts the normal gradient between intraocular pressure and retrolaminar pressure. This results in high tissue pressure within the optic nerves and further interrupts the metabolic processes that mediate axoplasmic flow, thereby causing papilledema (8). Visual symptoms and signs of papilledema range from minor, such as transient visual blur, arcuate scotomas, particularly beginning infero-nasally, to severe, such as blindness, depending on the degree of papilledema (9). Funduscopic examination often reveals an enlarged blind spot. The typical treatment to improve visual outcomes is surgical removal of the space-occupying lesion to relieve the IICP (8). Nonetheless, sometimes, the IICP may not be evenly distributed in the intracranial cavity, leading to asymmetric optic nerve injury. Another potential mechanism was introduced in a study by Ju et al., in which they found pre-operative tumor-related edema of the optic tract caused by sellar and suprasellar tumors could be the cause of worsened visual symptoms (10). In such a situation, atypical visual field defects may result from optic edema rather than the brain lesion itself.

We report a case of a young man with a convexity meningioma who initially presented with symptoms of right blurred vision and headache. His visual symptoms improved significantly following surgical removal of the tumor. We reviewed his magnetic resonance images (MRI) and hypothesized that the IICP caused by the large tumor led to the deviation of the pituitary stalk, resulting in an indentation on the right just-decussated nasal fibers and stretching of the right optic nerve; these factors contributed to his atypical visual symptoms.

## Case report

**Figure 1:**
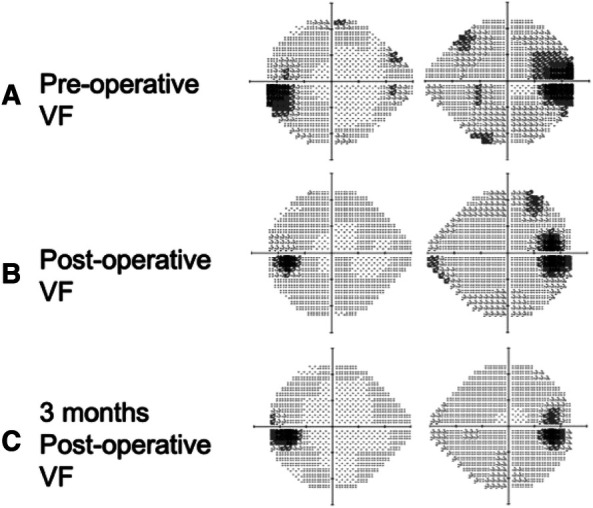
Humphrey visual field test pre-operatively, post-operatively, and 3 months post-operatively. (**A**) Pre-operative visual field test showing an enlarged blind spot bi-temporally (gray-to-dark region), (**B**) post-operative visual field test showing mild improvement in visual field defect compared to the pre-operative visual field test, and (**C**) a 3-month post-operative visual field test showing further improvement of the visual field.

**Figure 2:**
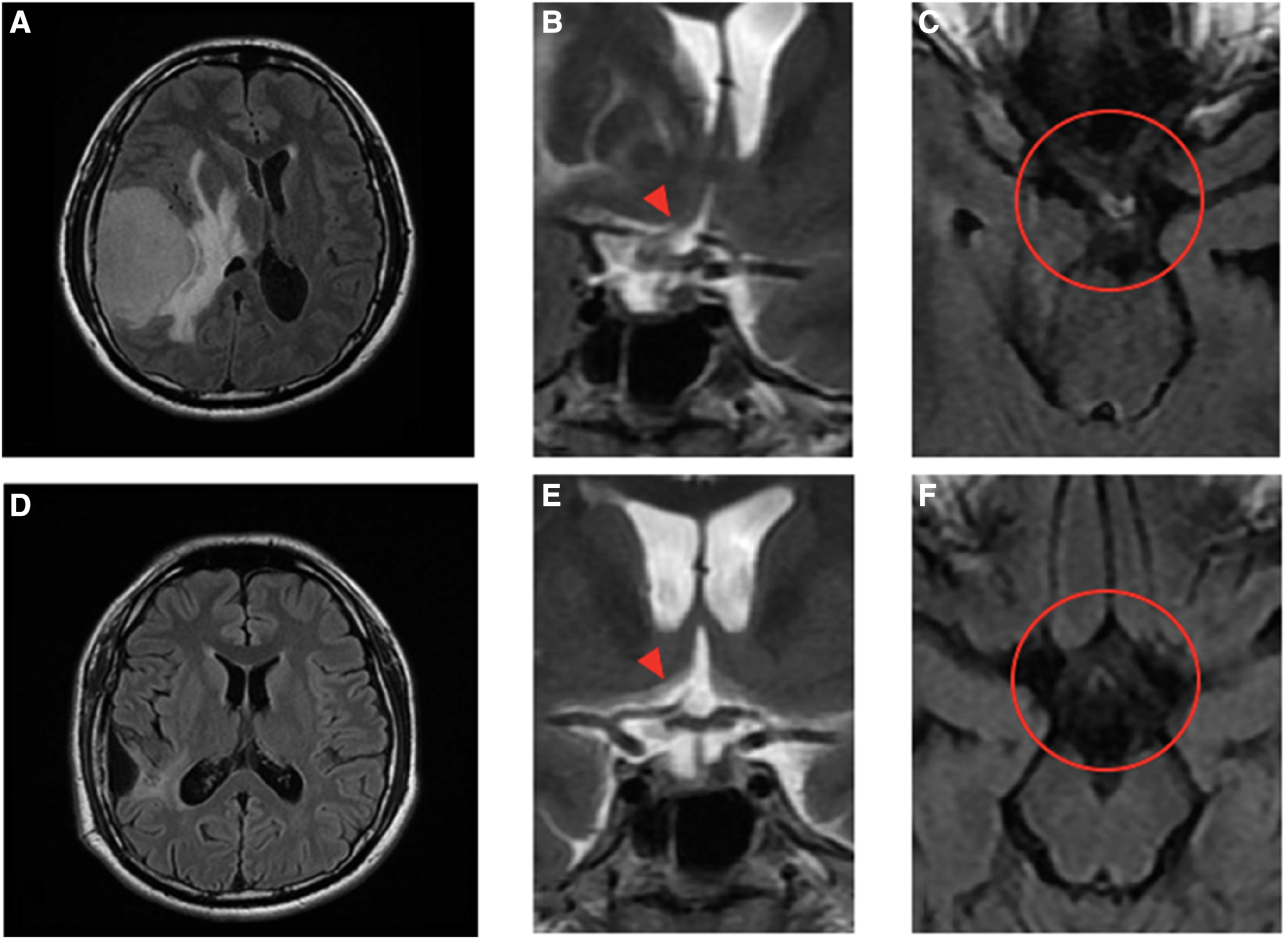
Pre- and post-operative MRI images and the magnified view of the optic chiasm in coronal and axial views. Pre-operative images (**A**)–(**C**) show a large extra-axial hypervascular tumor measuring 6.1 cm × 4.3 cm at the right temporoparietal convexity, causing a significant mass effect with a 1.3 cm leftward midline shift, uncal herniation, and midbrain compression. In the coronal view (**B**), the stalk and pituitary gland (arrowhead) are deviated to the left and forced against the optic chiasm, which is thicker. The edematous nerve fibers on the temporal side of the right optic nerve and the nasal side of the left just-decussated fibers can be clearly seen in the T2-weighted FLAIR MRI image, as shown in the circle in (**C**). Post-operative images (**D**)–(**F**) show no tumor and normalization of midline structures. The optic chiasm and pituitary stalk (arrowhead), as well as the third ventricle and hypothalamus, all remain in the central position and have no compressive effect (**E**). There is no edema along the optic chiasm to tract on pre- and post-chiasmatic nerve fibers, as shown in the circle in (**F**).

**Figure 3:**
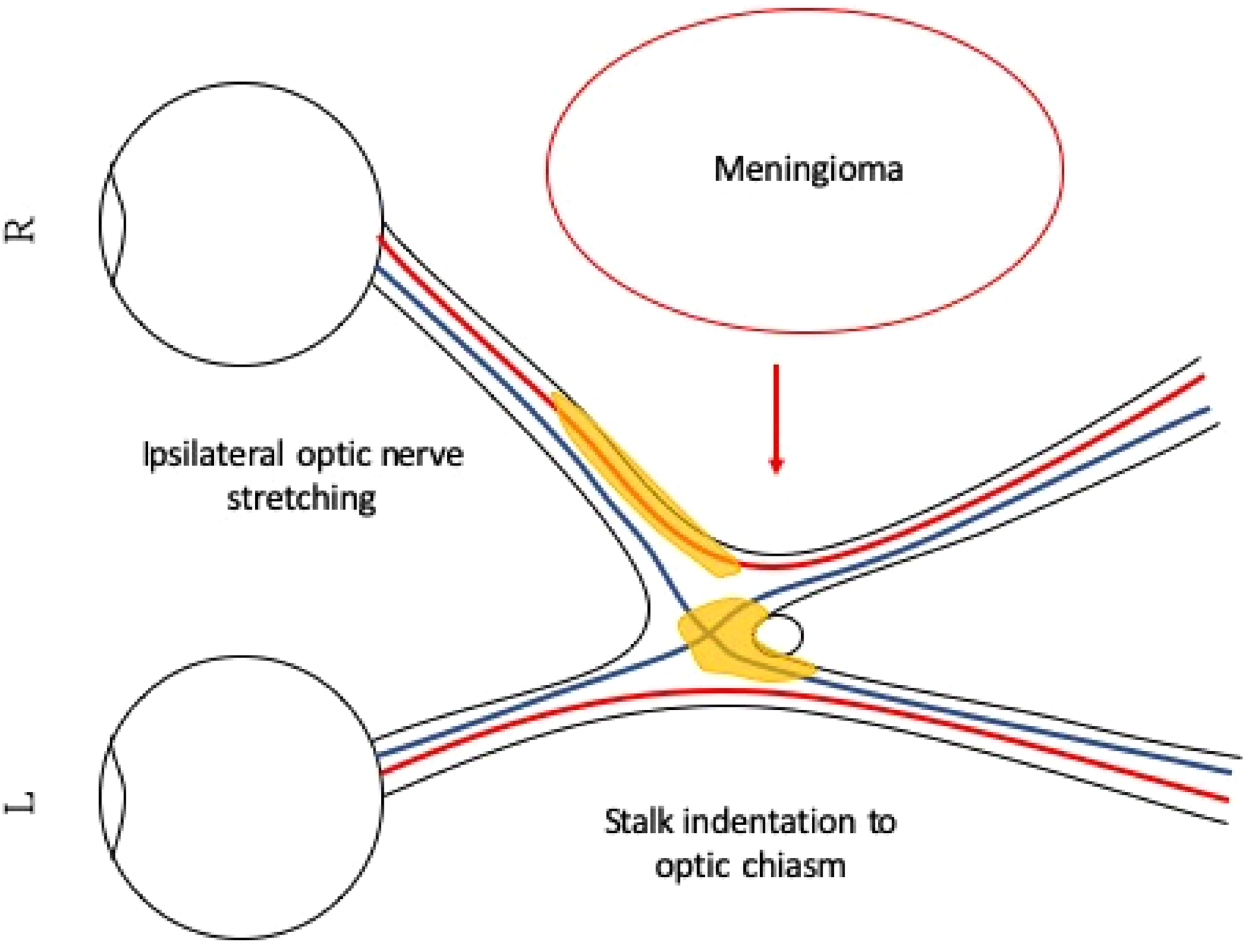
Illustration of right temporal optic nerve fiber stretching and pituitary stalk indentation on the right just-decussated nasal fibers. The large right parietal meningioma is pushing the optic apparatus to the left, causing the right optic nerve to stretch. The pituitary stalk is also pushed to the left, indenting on the optic chiasm. The stretched right optic nerve and the compressed optic chiasm show signs of injury, and we can see edematous changes on the MRI T2-weighted image. The edematous area is illustrated in yellow. The right temporal optic nerve fibers and the just-decussated right nasal optic fibers are injured, and together, they demonstrate clinically atypical visual field defects.

A 32-year-old man with a history of hypertension presented to our ophthalmic clinic with right blurred vision and intermittent headache for 1 week. He denied experiencing fever, nausea, vomiting, or head injury. Neurological examination revealed no focal deficits, except for a decline of visual function. The visual acuity was 20/40 in the right eye (OD) and 20/125 in the left eye (OS). Funduscopic examination revealed bilateral optic nerve swelling, optic disc edema, and hemorrhage. Humphry visual field examination demonstrated an asymmetrically enlarged blind spot bilaterally (Figure 1A). A T1-weighted contrast-enhanced MRI revealed a large extra-axial hypervascular tumor measuring 6.1 cm × 4.3 cm at the right temporoparietal convexity, causing significant mass effect with a 1.3 cm leftward midline shift, uncal herniation, and midbrain compression, indicative of significant IICP (Figure 2). Furthermore, we noted that the right optic nerve was deviated to the left with evident stretching and edema, especially affecting the temporal fibers, as indicated by high signal intensity on T2-weighted images. In addition, the pituitary stalk was deviated to the left, causing focal indentation on the just-decussated nasal fibers of the optic chiasm (Figure 2C). An illustrated image is shown in Figure 3. Subsequently, the patient was admitted to the neurosurgery ward for surgical intervention.

We arranged for pre-operative trans-arterial embolism (TAE) to de-vascularize the tumor pre-operatively. The craniotomy for tumor resection was performed on the following day. Pathological analysis revealed an atypical meningioma (World Health Organization grade 2) with necrosis, indicating a relatively faster growth rate than grade I meningiomas. The blurred vision of the patient rapidly improved in the days following surgery. A follow-up visual field examination 1 week after the surgery showed improvement compared to the pre-operative visual field (Figure 1B).

A 3-month MRI scan indicated complete removal of the tumor with normalization of the compressive effects on the optic apparatus (Figures 2D–F). Visual field examination demonstrated further improvement in bilateral visual fields, with a reduced size of blind spots (Figure 1C). Optical coherence tomography revealed a decrease of the averaged thickness of the retinal nerve fiber layer bilaterally, with improvement in the right eye from 192 to 142 μm and in the left eye from 174 to 112 μm at the 3-month follow-up, indicating a decrease in intracranial pressure.

## Discussion

IICP has been identified as a potential cause of visual field defects in hydrocephalic patients (11, 12). These visual defects can include quadrantanopia, bilateral central scotoma, and bi-temporal hemianopia (11, 12). On the other hand, Taxel et al. reported an interesting case involving the development of a new visual defect characterized by left parafoveal and peripheral field defect 8 months after bromocriptine treatment for a pituitary macroprolactinoma. In their report, the MRI showed downward and leftward traction and stretching of the optic chiasm due to marked shrinkage of the tumor, which has been proposed as the probable mechanism for the new visual defect (13). The combination of the above mechanisms may support our hypothesis according to the MRI findings, meaning a combined effect of: (1) IICP induced by an enlarging tumor causing significant deviation of midline structures; and (2) stretching of the optic nerve with nerve edema and indentation of the pituitary stalk on the optic chiasm, causing atypical visual deterioration in our patient.

Studies have reported visual field improvement after surgery for tumors in the sellar region. Between 42% and 88% of patients with suprasellar meningioma who underwent surgery showed visual improvement (14–17). Similar results were seen in patients with pituitary adenoma after endoscopic endonasal transsphenoidal surgery, with visual improvement noted in 80.8% of these patients (18). Also, visual improvement after surgery has been documented in patients with tumors or lesions located outside the sellar region. Xiao et al. reported improvement in visual symptoms and papilledema in patients with ventriculomegaly after surgical treatment with ventriculostomy to relieve the IICP (19). In addition, visual symptoms improved in 70% of the patients with occipital lobe metastasis after a combination of surgery and radiation therapy (20). Galal et al. identified indicators that predict a better visual outcome post-operatively, including duration of symptoms less than 1 year, partial visual deficits, tumor size less than 3 cm in maximum diameter, and a lesser degree of stretch and compression of the optic apparatus (17).

Interestingly, pre-operative TAE has also been reported to benefit visual recovery for hypervascular tumors (21). The advantage of reducing vascularization before and during tumor resection is obvious. Therefore, for both sellar and extrasellar tumors, a careful evaluation of feeding vessels that do not compromise optic nerve blood supply may suggest embolization to facilitate tumor removal and enhance visual recovery.

When encountering a patient with an extra-axial brain tumor located distant from the optic pathway, there remains a possibility of optic symptoms occurring. In such cases, it is essential to perform a pre-operative evaluation of brain MRI to assess for pituitary stalk indentation and optic nerve stretching. In addition, conducting visual field examinations and seeking ophthalmology consultation are crucial steps. In this case, we observed significant improvement in visual symptoms following surgical treatment of the remote brain tumor. Prompt diagnosis and surgical intervention are crucial for achieving complete recovery of visual symptoms. On the other hand, if a distant tumor is left untreated, the visual symptoms may worsen. Further clinical studies and research are necessary to substantiate the above assertions.

In summary, brain tumor-induced IICP leading to visual disturbance, whether directly or indirectly, is not uncommon. However, extrasellar extrinsic brain tumors causing atypical visual field defects are rarely reported. Such atypical defects may result from asymmetric pressure on the optic apparatus surrounding the sellar region due to a deviation of cerebral midline structures. The pituitary stalk, located immediately behind the optic chiasm, may induce focal pressure and worsen visual signal transmission along the optic pathway. By presenting this case, we have demonstrated that a potential combined mechanism of nerve stretching with edematous changes and pituitary stalk indentation, despite rare, could clearly be identified on MRI. By illustrating the relationship between the stalk and the optic chiasm, we aim to emphasize that stalk indentation might have a role in aggravating visual defects in patients with space-occupying lesions localized in the sellar or extrasellar regions.

## Data Availability

The original contributions presented in the study are included in the article/Supplementary Material, further inquiries can be directed to the corresponding author.
